# Trends in Antimicrobial Prescription for Inpatients in Changsha, China, 2003 to 2014

**Published:** 2017-09

**Authors:** Yinhua ZHANG, Qun YUAN, Xia YI, Honghua LIU, Xiaoyan PAN, Jingwei LIU, Yi XU, Yang CHEN, Guoping HE

**Affiliations:** 1. Dept. of Community Nursing, School of Nursing, Central South University, Changsha, P.R. China; 2. Dept. of Fundamentals of Nursing, School of Nursing, Hunan University Traditional Chinese Medicine, Changsha, P.R. China; 3. Medical Insurance Bureau of Hunan Province, Changsha, P.R. China

**Keywords:** Antimicrobial, Prescription, Inpatient, China

## Abstract

**Background:**

China had implemented policies to limit antimicrobials prescription since 2004; we conducted this study to reflect the effect of these national policies by analyzing antimicrobial prescription trends of medical insurance in patients from 2003 to 2014 in Changsha city, China.

**Methods:**

The participants were inpatients of the medical insurance of urban workers (UEBMI). Data were extracted from medical insurance information system of Changsha Medical Insurance Institution, which directly connects with hospitals information systems.

**Results:**

Trend analysis showed great changes in antimicrobial prescription and inpatients’ cost on antimicrobials over the study period. Antimicrobial prescription rates gradually declined over the study period from 79.0% in 2003 to 43.5% in 2014 (adjusted OR0.205; 95%CI 0.198 to 0.213). There was a quicker decline from 2011 to 2014 (with implementing national antimicrobial stewardship action plan) than the period from 2003 to 2010 (with implementing antimicrobials use education and self-management strategies). The proportion of inpatients used one antimicrobial increased significantly from 25.6% in 2003 to 46.7% in 2014, while the proportion of inpatients used three or more antimicrobials gradually decreased. Bacterial culture rate increased from 20.4% in 2003 to 36.6% in 2014 (adjusted OR 2.248; 95% CI 2.149 to 2.352). The average costs on antimicrobials decreased significantly, from 277.43 US Dollar in 2003 to 91.05 US Dollar in 2014.

**Conclusion:**

National efforts to promote rational use of antimicrobials in clinical practice have had a positive effect over the past decade in China.

## Introduction

Antimicrobial resistance is one of the biggest challenges to global public health today (1–3), which may prolong length of stay in hospital, rise medical expenditures and threaten global health. Antimicrobial resistance in China is higher than in western countries and the prevalence of resistant bacteria appears to be a striking increase (4).

Antimicrobial resistance occurs naturally, inappropriate use of antimicrobial is considered as one of the important causes of accelerating the process (5, 6). Inappropriate use of antimicrobials is a serious problem in China, 68.9% of inpatients have used antimicrobials; among them 37% of inpatients have used two or more antimicrobials in China in 2010 (7).

Antimicrobial stewardship program (ASPs) have been implemented worldwide to expect to reduce antimicrobial overuse and misuse and combat antimicrobial resistant pathogens, as the large pharmaceutical market of the world’s most populous country, China is no exception (8). China’s first antimicrobial guideline--Guidelines for antimicrobial use in clinical practice was issued by the Ministry of Health of China in 2004, to control antimicrobial prescription rate and discourage inappropriate use of antimicrobial. In 2011, 2012, 2013, the Ministry of Health of China initiated national antimicrobial stewardship action plan in combating antimicrobial resistance (9–11). Few long-term studies have been conducted to examine the effects of introduction of the policy to prevent abuse of antimicrobials in China. This study aimed to assess the impact of these national policies on antimicrobial use by identifying and reporting on antimicrobial use trends in insured inpatients in Changsha, South China from 2003 to 2014.

## Methods

### Participant’s source

This observational retrospective study was conducted in Changsha City, the capital of Hunan Province, South China. This city has a population of 3.04 million, birth rate 15.3% and mortality rate of 5.3%. The number of hospitals beds increased from 24300 in 2003 to 63600 in 2014. The socioeconomic status, demographics, resident income, and population health are similar to other cities in South China. To some extent, this region is representative of major urban areas in South China.

Medical insurance for urban workers (UEBMI) began in 2000 in Changsha City; the insured persons are current and retired workers. Employers and employees pay the insurance premium together. The insured persons are 1.81 million, nearly accounts for 59.5% of the total population in Changsha City. The ages of all insured persons are over 18 yr old and the retiree proportion was approximately 28%. UEBMI in Changsha City is mandatory. The insured persons are relatively fixed and generally did not exist unless finding a job in another city or on death, and are engaged in two medical insurance institutions. One medical insurance institution includes approximately 80% of insured persons and another institution includes approximately 20%.

The data in this study were derived from the Second Medical Insurance Institution, which includes 210–360 thousand insured persons from 2003 to 2014, accounting for approximately 10.7%–11.8% of Changsha population.

### Data collection and outcome evaluation

The participants were insured inpatients Changsha City from 2003 to 2014. The inpatients were defined as those who received treatment for more than 1 day in hospital wards. Data were extracted from medical insurance information system of the second medical insurance institution of Changsha city. The Medical insurance information system directly connects with the hospital information systems. To ensure that the data are in complete accord with hospital records, quality control of the data is maintained through daily authenticity evaluation and randomized data checking. Daily authenticity evaluation refers to medical insurance personnel verifying the data consistency of the medical insurance information system and hospital information systems daily; 5%–10% of all inpatients’ medical records were checked during the study period. The Medical Insurance Institution of Changsha City pays hospitalization costs based on the data in the information system. Therefore, the data of this study were accurate and reliable. Randomized data checking refers to researchers verifying random samples of inpatient medical information from medical records against the medical insurance information system; 1% of inpatients records were checked in this manner. Data included inpatients’ antimicrobial prescriptions (the type, number and administration route of antimicrobial prescriptions), bacterial culture and hospitalization cost (total hospitalization cost, costs on medication and cost on antimicrobial).

Antimicrobials in this study refer to antimicrobials and synthetic antimicrobials according to the WHO ATC group J01. We identified antimicrobial prescriptions by both trade and generic names. Antimicrobial prescription rate was determined as the proportion of inpatients prescribed antimicrobials accounted for total inpatients. Injected antimicrobial prescription includes intramuscular injection, intravenous injection and intravenous infusion. Combined antimicrobial prescription refers to the use of two or more antimicrobials at the same time during a hospitalization period. Bacterial culture rate is defined as the proportion of inpatients whose specimens were received bacterial culture accounted for the inpatients who administered antimicrobial for the purpose of therapy.

### Statistical analysis

Data were entered into SPSS 19.0 (Chicago, IL, USA) and subsequently subjected to statistical analysis. Descriptive statistics, chi-square statistics and trend analysis were used in this study. Rates were compared between different years. Risk ratios and 95% confidence intervals (95% CIs) were calculated. Significance of trends was based on a weighted least-squares regression analysis at the *P*<0.01 level of confidence.

The study was approved by the Medical Insurance Bureau Board of Changsha City and Medical Ethical Committee at Hunan University of Traditional Chinese Medicine.

## Results

### Insured inpatients and hospitals

The number of insured inpatients was 18505 in 2003, and it increased to 92426 in 2014. The number of involved hospitals also have been an increasing trend, there were 57 hospitals involved in 2003 and 111 hospitals in 2014. Insured inpatients and involved hospitals are shown in Table 1. Hospitals include comprehensive hospitals, specialty hospitals (i.e. maternity hospitals, children’s hospitals, cancer hospitals, infectious disease hospital).

**Table 1:** The number of insured persons, insured inpatients, antimicrobial prescription and hospitalization costs

**Year**	**Insured persons**	**Insured inpatients (%)**	**Number of involved hospitals**	**Antimicrobial prescription Rate (%)**	**Bacterial culture rate (%)**	**Combined antimicrobial prescription (%)**			**Administration route of antimicrobial proscriptions (%)**		**Inpatients’ cost (US Dollar)**		

						**One**	**One**	**One**	**oral**	**injected**	**Total hospitalization cost**	**Cost on medication**	**Cost on antimicrobials**
**2003**	214700	18505(8.6)	57	79.0	20.4	25.6	27.3	47.1	35.7	73.9	314.8	763.4	277.4
**2004**	221500	22975 (10.4)	64	74.1	24.7	28.5	28.8	42.7	28.4	69.6	1454.8	677.3	243.4
**2005**	230700	27823(12.1)	66	73.5	27.7	27.9	27.8	44.4	30.6	69.0	1367.7	678.9	242.2
**2006**	241700	31256(12.9)	76	72.8	31.9	26.4	27.1	46.5	36.3	67.6	1373.6	668.2	223.6
**2007**	280600	39144(14.0)	88	68.8	30.6	28.5	27.9	43.6	33.8	63.6	1383.2	687.1	236.1
**2008**	302000	45971(15.2)	92	66.2	29.8	29.7	29.1	41.1	32.8	60.9	1385.8	782.6	269.7
**2009**	312900	53945(17.2)	95	66.3	29.5	29.3	29.8	40.9	38.8	59.2	1523.1	795.3	275.5
**2010**	334791	62088 (18.5)	99	65.1	27.4	30.7	30.6	38.6	37.2	57.3	1551.7	829.4	167.4
**2011**	351655	72125(20.5)	95	58.3	27.9	35.3	30.3	34.4	26.7	52.9	1639.4	814.7	117.9
**2012**	347321	77821(22.4)	90	48.8	32.7	41.5	30.2	28.3	18.1	44.9	1708.3	869.6	88.1
**2013**	354699	87633(24.7)	106	44.0	34.5	44.5	29.7	25.8	15.8	40.3	1827.7	913.8	81.6
**2014**	365877	92426(25.3)	111	43.5	36.6	46.7	29.1	24.2	15.3	39.9	1938.8	938.9	91.0
**χ^2^**				34993.62	2489.64		11706.48		9855.41	1317.99			
***P***				0.000	0.000		0.000		0.000	0.000			

### Antimicrobial prescription rate

**Fig. 1:**
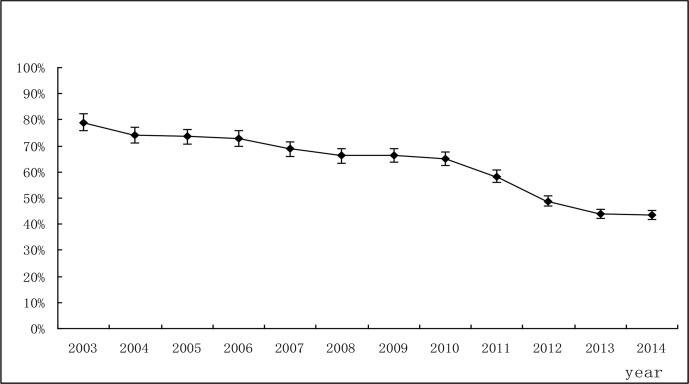
Trends in annual antimicrobial prescription rate of inpatients -Changsha city, China, 2003–2014 -Error bars indicate 95% confidence intervals Rates are per 100 inpatients-Note: All trends shown are significant (χ^2^=34993.62, *P*<0.001)

Annual antimicrobial prescription rates are shown in Table 1. Antimicrobial prescription rates gradually declined over the study period from 79.0% in 2003 to 65.1% in 2010 (adjusted OR0.496, 95% CI 0.477 to 0.516), then rapidly fell from 65.1% in 2010 to 43.5% in 2014 (adjusted OR0.414; 95% CI 0.405 to 0.423) (Table 1) (Fig. 1).

### Combined antimicrobial prescription

**Fig. 2:**
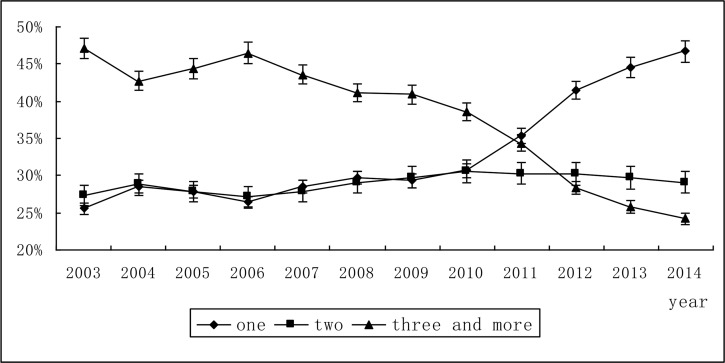
Trends in the proportion of single or combined antimicrobial administration of inpatients -Changsha city, China, 2003–2014. -Error bars indicate 95% confidence intervals. Rates are per 100 in-patients who used antimicrobials- Note: All trends shown are significant (χ^2^=11706.48, *P* <0.001).

Among those inpatients with antimicrobials prescribed, the proportion of inpatients used one antimicrobial increased significantly from 25.6% in 2003 to 46.7% in 2014, the proportion of inpatients used two antimicrobials increased mildly from 27.3% to 29.1%. Meantime, the proportion of inpatients used three or more antimicrobials gradually decreased from 47.1% to 24.2% (Table 1, Fig. 2).

### Administration route of antimicrobial prescriptions

Oral antimicrobial prescription rates increased from 28.4% in 2004 to 37.2% in 2010 (adjusted OR 1.493; 95% CI 1.444 to 1.543), then reduced annually, to 15.3% by 2014 (adjusted OR 0.306; 95% CI 0.299 to 0.314). Injected antimicrobial prescription rates steadily declined from 73.9% in 2003 to 39.9% in 2014 (adjusted OR 0.234; 95% CI 0.226 to 0.242) (Table 1).

### Bacterial culture rate

Among inpatients administered antimicrobial for the purpose of therapy, bacterial culture rate experienced a growth trend during the study period, from 20.4% in 2003 to 36.6% in 2014 (adjusted OR 2.248; 95% CI 2.149 to 2.352) (Table 1).

### Inpatients’ cost

The inpatients’ average total hospitalization cost presents an increasing trend. The average costs of medication show a mild increasing during the study period. The average costs on antimicrobials decreased significantly, from 277.43 US Dollar in 2003 to 91.05 US Dollar in 2014 (Table 1).

### 2014 Antimicrobial prescription: different level hospitals

In 2014, total 111 hospitals were involved, 55 were primary care hospitals, 26 were secondary hospitals and 30 were tertiary hospitals. The highest antimicrobial prescription rate was found in primary care hospitals (53.5%) and the lowest rate was in tertiary hospitals (40.2%) (*P*=0.001). The proportion of inpatients used one antimicrobial for tertiary hospitals (52.6%) was higher (*P*=0.001) than that in primary care hospitals (31.4%) and secondary hospitals (46.7%), accordingly, the proportion of inpatients used two, three or more antimicrobials for tertiary hospitals (26.6% and 20.8%) was lower (*P*=0.002) than that in primary care hospitals (35.4%, 33.2%) and secondary hospitals (29.2%, 24.2%). The bacterial culture rate for the tertiary hospitals (29.6%) was higher than the primary care hospitals (3.9%) and the secondary hospitals (15.8%). The proportion of cost on antimicrobials accounted for cost of medication in primary care hospitals (39.2%) was higher than the secondary hospitals (28.3%) and the tertiary hospitals (24.6%). The same was observed for the proportion of cost on antimicrobials accounted for cost of total hospitalization, with the primary care hospitals (24.7%) having a higher proportion than the secondary hospitals (14.5%) and the tertiary hospitals (10.8%) (Table 2).

**Table 2:** 2014 Antimicrobial prescription: hospital classifications

**Hospital classification**	**involved hospitals**	**Antimicrobial prescription rate (%)**	**Bacterial culture rate (%)**	**Combined antimicrobial prescription (%)**			**Inpatients’ cost (US Dollar)**				
				**One**	**Two**	**Three or more**	**Total hospitalization cost**	**Cost on medication**	**Cost on antimicrobials**	**Cost on antimicrobials/Cost on medication (%)**	**Cost on antimicrobials/Total hospitalization cost (%)**
**Primary care hospital**	55	53.5	3.9	31.4	35.4	33.2	536.3	338.0	132.5	39.2	24.7
**Secondary hospital**	26	45.2	15.8	46.7	29.2	24.2	1106.8	565.3	160.6	28.3	14.5
**Tertiary hospital**	30	40.2	29.6	52.6	26.6	20.8	2912.7	1277.8	314.8	24.6	10.8
**χ^2^**		984.06	6206.28	1245.53							
***P***		0.000	0.000	0.000							

## Discussion

China had implemented policies to limit antimicrobials prescription since 2004, while the impact of national policies on rational use of antimicrobials has not been fully evaluated over the last decade. We conducted this study to reflect the effect of these national policies by analyzing antimicrobial prescription trends of medical insurance inpatients from 2003 to 2014 in Changsha City, South China. Data were extracted from medical insurance information system and trend analysis was conducted in this study.

The antimicrobial prescription rate is a key indicator for assessing whether the use of antimicrobial is appropriate (12). In this study, antimicrobial prescription rates showed a significant decline during study period, from 79.0% in 2003 to 43.5% in 2014. This result is consistent with the findings (13, 14). Antimicrobial use prevalence was decreased from 54.79% in 2001 to 46.63% in 2010 by surveying tertiary hospitals from all 31 provinces of China. The average antimicrobial prescribing rate was reduced significantly from 62.9% in 2010 to 35.3% in 2014 in 65 public general hospitals of China.

This study also proves that government intervention has been regarded as an effective method for ensuring rational use of antimicrobials on the hospital level (15–18). With 2011 as the time node, different decline speed of antimicrobial prescription rates was presented. There was a larger decrease from 2011 to 2014 (65.1%–43.5%, on average decreased by 5.4% annually) than in the period from 2003 to 2010 (79.0%–65.1%, on average decreased by 2.0% annually). We think the reason is that two different antimicrobial stewardship strategies were conducted by the Ministry of Health of China during the two periods. Antimicrobials use education and self-management strategies were implemented from 2004 to 2010. By taking a series of measurements, such as issuing guidelines for antimicrobial use in clinical practice, conducting clinicians’ education of antimicrobials use, supervising the antimicrobial prescriptions by the Infection Control Committee of hospitals, antimicrobial stewardship has been effective, antimicrobial prescription rates declined from 79.0% (2003) to 65.1% (2010) during 7 yr period, but have not achieved the expected goals; antimicrobial prescription rates were still relatively high in 2010. As a combination of professional and administrative strategies, national antimicrobial stewardship action plan was initiated in 2011, 2012 and 2013 (9–11), including the following measurements: 1) continuously reinforcing antimicrobial treatment guideline and strengthening clinicians’ antimicrobials use education. Only those clinicians who received antimicrobials use training and passed the exam can obtain the right to prescribe antimicrobials. 2) setting the antimicrobials stewardship goals, such as limited antimicrobial prescription rate for inpatients and outpatients to be less than 60% and 20% respectively in general hospitals, controlled Antimicrobial Use Density (AUD) to be under 40 DDD per 100 bed-days in general hospitals. 3) According to antimicrobials classification stewardship regulation, antimicrobials are divided into three grades: non-restricted, restricted and special-grade. The authority to prescribe different grades of antimicrobial is determined by clinician’s professional title. 4) The antimicrobial prescription peer review process has been implemented. Twenty-five percent of all antimicrobial prescriptions should be peer-reviewed and more than 50 antimicrobial prescriptions for every clinician should be peer reviewed every year. 5) Penalties were used. The chief administrators of the hospital are the first responsible person for rational use of antimicrobials. Clinicians whose use of antimicrobials is unreasonable could be criticized and even have prescription authority revoked.

Oral antimicrobial prescription rates increased annually from 2004 (28.4%) to 2010 (37.2%). Guidelines for antimicrobial use in clinical practice, issued by the Ministry of Health of China in 2004, provided the following direction: oral antimicrobials should be used for patients with mild infections; antimicrobials should only be administrated by injection route for patients with severe infections or systemic infections. When patients’ infections resolve, injected antimicrobials should be replaced by oral antimicrobials. The clinicians tended to prescribe oral antimicrobials once injected antimicrobials are stopped, in order to maintain the therapeutic effect. However, oral antimicrobial prescription rates reduced annually from 2010 (37.2%) to 2014 (15.3%), the probable reason is that national antimicrobial stewardship action plan (9) (2011) required that AUD should be under 40 DDDs per 100 bed-days. In order to reach this AUD requirement, clinicians tried to reduce unnecessary oral antimicrobial use.

The present study found antimicrobial prescription rate, the proportion of cost on antimicrobials accounted for cost of medication and accounted for cost of total hospitalization were higher, the proportion of inpatients used one antimicrobial and bacterial culture rate was lower in primary care hospitals than in secondary hospitals and tertiary hospitals. The highest antimicrobial prescription rate was found in primary care hospitals. Hospitals in primary care centers tend to be associated with higher antimicrobial use than hospitals in high-level hospitals in China (6). Antimicrobial consumption decreased with increased hospital size (19). It is reasonable to infer that secondary hospitals and tertiary hospitals have a better performance in rational use of antimicrobial, compared with primary hospitals. Secondary hospitals and tertiary hospitals were mandatory to participate in national antimicrobial stewardship action plan by the Ministry of Health, there were stricter regulation of antimicrobial use in secondary hospitals and tertiary hospitals than in the primary hospitals. With better medical education background and more knowledge of antimicrobial rational use, the clinicians in secondary hospitals and tertiary hospitals have stronger awareness of cautious use of antimicrobial. Chinese government should strengthen antimicrobial stewardship strategy focusing on primary hospitals to promote the rational use of antimicrobial in primary hospitals in China.

There are limitations to this study. Firstly, there was no antimicrobial use data on some special diseases such as appendicitis and pneumonia, which can illustrate antimicrobial use comprehensively and effectively. Further studies focused on inpatients with some specific diseases. In addition, we have not analyzed the AUD and antimicrobial species, which are also important indicators of evaluating the rationality of antimicrobial use.

## Conclusion

National efforts to promote rational use of antimicrobials in clinical practice have had a positive effect over the past decade in China. With antimicrobial prescription rate, combined antimicrobial prescription rate and costs on antimicrobials significantly decreased, bacterial culture rate increased, antimicrobials use has become rational gradually.

## Ethical considerations

Ethical issues (Including plagiarism, informed consent, misconduct, data fabrication and/or falsification, double publication and/or submission, redundancy, etc.) have been completely observed by the authors.
